# Oxidative Stress and Inflammation in Cardiovascular Disease

**DOI:** 10.1155/2017/5853238

**Published:** 2017-04-27

**Authors:** Noemí García, Cecilia Zazueta, Leopoldo Aguilera-Aguirre

**Affiliations:** ^1^Escuela de Medicina, Tecnológico de Monterrey, Monterrey, NL, Mexico; ^2^Departamento de Biomedicina Cardiovascular, Instituto Nacional de Cardiología-Ignacio Chávez, Ciudad de México, Mexico; ^3^Department of Microbiology and Immunology, University of Texas Medical Branch, Galveston, TX, USA

Evidence from experimental and clinical studies have shown that oxidative/nitrosative stress and inflammation associated with metabolic disorders such as obesity, hypertension, and diabetes conduce to left ventricular hypertrophy, fibrosis, diastolic dysfunction, heart failure, and ischemia/reperfusion damage [1, 2].

In this special issue, original researches on the causative effect of oxidative stress and inflammation on pathologies such as mitral valve prolapse (MVP), arteriosclerosis, hypertension, and ischemia/reperfusion (IR) injury are presented. Also, the evidence of the effect of NLRP3-inflammasome and reactive oxygen species (ROS) production by Ca2+ overload on mitochondrial function, along with the demonstration of the regulatory properties of MG132, demetoxicurcumin, and lycopene on signaling pathways that activate either nuclear factor E2-related factor 2 (Nrf2) or NF*κ*B, are shown. Information in this issue also includes findings on the relationship between ROS and the activation/maturation of dendritic cells (DCs) that might trigger cardiovascular and metabolic pathologies.

The strong association between osteoprotegerin (OPG) levels and oxidative stress status in patients affected by MVP with severe regurgitation opens its possible use as a serum marker of this pathology according to P. Songia et al. On the other hand, R. Mastrocola et al. demonstrate that the inhibition of NLP3-inflammasome reduces IR injury by activating prosurvival RISK pathway and preserving mitochondrial function. This is interesting and is related to the article presented by Y. Oropeza-Alamazán et al., which demonstrates that silencing of MCU decreases ROS production and mitochondrial dysfunction induced by Ca2+ overload in reperfusion injury.

Pharmacological regulation of oxidative stress and inflammation is also included in this special issue. Y. Li et al. demonstrated that the downregulation of cyclooxygenase (COX-2) by demethoxycurcumin (DMC) favors nitric oxide (NO) production via e-NOS activation, preventing endothelial dysfunction, whereas L. Kong et al. demonstrated that MG132 administration inhibits Nrf2 and IkB proteolysis via the proteasome, preventing endothelial dysfunction associated with cardiovascular disease in animals with diabetic nephropathy. The antioxidant and anti-inflammatory properties of lycopene are shown to be related with the downregulation of Rho-associated kinases and the activation of key factor expression in the study described by Y. He et al., who propose that this compound might by a potentially effective method for transplant arteriosclerosis in clinical organ transplantation. Finally, J. Stein et al. show that Nox2-derivated oxidative stress and PKC activation play relevant roles in DCs activation and the atherothrombotic processes that might impinge on cardiovascular and metabolic pathologies.

We hope that the research articles included in this issue contribute to the understanding of mechanisms related to the development of cardiovascular diseases and increase the possibility to find novel specific markers and the proposal of new potential therapeutic treatment with the use of new drugs that regulated pathway risk related with the trigger of cardiovascular disease.

## References

[1] Sverdlov A. L., Elezaby A., Qin F. (2016). Mitochondrial reactive oxygen species mediate cardiac structural, functional, and mitochondrial consequences of diet-induced metabolic heart disease. *Journal of the American Heart Association*.

[2] Yao X., Carlson D., Sun Y. (2015). Mitochondrial ROS induces cardiac inflammation via a pathway through mtDNA damage in a pneumonia-related sepsis model. *PLoS ONE*.

